# Evaluating the Ecological Conditions of a Semi-Arid River Basin: A Multimetric Index Incorporating Fish and Macroinvertebrate assemblages

**DOI:** 10.1007/s00267-025-02319-7

**Published:** 2025-11-28

**Authors:** Mojgan Zare Shahraki, Ali Reza Esmaeili Ofogh, Pejman Fathi, Joseph Flotemersch, Karen Blocksom, Andreas Bruder

**Affiliations:** 1 Institute of Microbiology, University of Applied Sciences and Arts of Southern Switzerland, Mendrisio, Switzerland; 2 FWBON the Freshwater Biodiversity Observation Network of GEOBON, Montreal, QC Canada; 3 U.S. Environmental Protection Agency, Office of Research and Development, Ohio, OH USA; 4 U.S. Environmental Protection Agency, Office of Research and Development, Oregon, OR USA

**Keywords:** Bioindicators, Ecosystem health, Freshwater ecosystem, Anthropogenic disturbances

## Abstract

The health and integrity of freshwater ecosystems are significantly affected by anthropogenic pressures. Understanding the ecological conditions of freshwater ecosystems is crucial for effective conservation and management strategies. In this study, we developed a new multimetric index, the Karun Fish-Macroinvertebrate Index (KFMI), that incorporates data on fish and macroinvertebrate assemblages to assess the ecological conditions of the Karun River basin, in Iran. We sampled 53 sites and collected data on fish and macroinvertebrate communities, physicochemical parameters, and habitat characteristics. We used physicochemical and physical habitat characteristics data to identify reference conditions using the concept of least-disturbed condition and based on Principal Component Analysis (PCA). We calculated 54 fish and 363 macroinvertebrate metrics to represent different aspects of ecosystem health. We created multiple KFMIs by combining specific core metrics through a stepwise process that assessed metric stability, responsiveness to environmental variables, and redundancy. The final KFMI consisted of seven metrics (3 fish and 4 macroinvertebrate) related to taxa richness, community composition, functional diversity indices, functional feeding groups, reproduction status, and habitat preferences. The index showed good discrimination efficiency (92%) and precision in classifying sites into different ecological health categories and highlights the value of incorporating multiple biological assemblages in multimetric indices to support ecosystem assessment and management strategies.

## Introduction

Anthropogenic pressures have adversely affected biodiversity, ecosystem functions, and the various ecosystem services provided by freshwaters (Bănăduc et al. 2022; Reid et al. 2019; Tickner et al. 2020). Therefore, assessing ecosystem conditions, including water quality, physical habitat characteristics, and the biological condition of freshwater systems, is important for successful conservation frameworks and management practices (Lyons et al. 1995; Lainé et al. 2014; Herman and Nejadhashemi 2015). One approach is to evaluate the condition of aquatic ecosystems by monitoring biotic communities as indicators of river health, potentially including micro-organisms such as algae and protozoa, and macro-organisms like aquatic plants, fish, macroinvertebrates, and mammals (Suter and Cormier 2015; Herman and Nejadhashemi 2015). The combination of presence, absence, relative abundance, autecology, and community ecology of aquatic biota indicates the effects of various disturbances and environmental parameters that are not efficiently detected by analyses of chemical conditions alone (Reynoldson and Metcalfe-Smith 1992). Consequently, biological indicators of condition have been widely used in ecosystem monitoring and evaluation programs (Li et al. 2016; Curtean-Bănăduc et al. 2019; Ruaro et al. 2024).

The Index of Biological Integrity (IBI), introduced by Karr (1981), was the first multimetric index (MMI) used to evaluate stream health and function. Today, MMIs are frequently used in assessing freshwater ecosystem condition (Akamagwuna et al. 2022; Cao et al. 2007; Carvalho et al. 2017; Chen et al. 2019; Esmaeili Ofogh et al. 2024, 2023; Zare Shahraki et al. 2022b). MMIs are considered more robust than single-metric evaluation techniques or water quality assessment because multiple metrics have the potential to better detect and inform on the impacts of various factors causing ecosystem deterioration (e.g., altered flow regimes, organic pollution, nutrients, stream geomorphological changes, acidification) (Hering et al. 2006; Freund and Petty 2007; Lewin et al. 2014; Reyes-Celis et al. 2025).

Multi-metric Indices vary depending on the type of biotic assemblage, habitat considered, and regional characteristics (Johnson 2014; Shen et al. 2024). Fish, macroinvertebrates, and periphyton assemblages are most commonly used in aquatic bioassessment programs and thus in MMIs (USEPA 1986; Barbour et al. 1999; Shen et al. 2024). These assemblages are sometimes used together but assessed separately, as they vary in their sensitivity to different disturbances and in their resilience to physicochemical stressors (Townsend and Hildrew 1994; Griffith et al. 2005; Carlisle et al. 2008; Fierro et al. 2017). Most regulatory entities in the United States use multiple biotic assemblages (USEPA, 2024) per EPA recommendations (USEPA 1990; Gibson and Barbour 1996; Barbour et al. 1999). However, incorporating metrics of different assemblages into a single evaluative index of biological integrity could be an effective approach to detecting general environmental disturbances (Griffith et al. 2005; Clapcott et al. 2014; Mendes et al. 2014; Li et al. 2016; Chen et al. 2017; Ruaro et al. 2019). To advance bioassessment, the potential to combine mixed metrics needs to outweigh the possible disadvantages of combining assemblages, such as masking the effects of specific stressors (Carlisle et al. 2008; Ruaro et al. 2020; Vadas et al. 2022).

The development of MMIs in arid and semi-arid regions has been limited because of insufficient available data and challenges in their development; however, there have been recent developments in semi-arid regions (Hooper et al. 2005; Vander Laan and Hawkins 2014; Zare Shahraki et al. 2022b; Kaboré et al. 2022; Esmaeili Ofogh et al. 2023, 2024). We aimed to evaluate whether integrating fish and macroinvertebrate metrics into a single multimetric index would provide a more sensitive and holistic tool for detecting environmental degradation than single-assemblage indices using the Karun Basin, Iran as a case study (Zare Shahraki et al. 2022b; Esmaeili Ofogh et al. 2024). A combined index has the potential to capture different responses to environmental changes arising from the ecological differences between fish and macroinvertebrates (Flotemersch et al. 2006; Barbour et al. 1999). Fish assemblages reflect large-scale disturbances such as connectivity loss, flow alteration, and invasive species through their longer lifespans and greater mobility, while macroinvertebrate assemblages capture reach-scale changes in water quality and habitat with finer temporal resolution (Paller et al. 2014; Feio et al. 2023). We hypothesized that a combined index integrating fish and macroinvertebrate metrics provides a more robust assessment of ecological condition than indices based on either group alone. Building on this rationale, we developed the Karun Fish and Macroinvertebrate Index (KFMI) as a comprehensive tool to evaluate ecological integrity in the Karun Basin and beyond.

## Materials and Methods

### Study Area

**Fig. 1:**
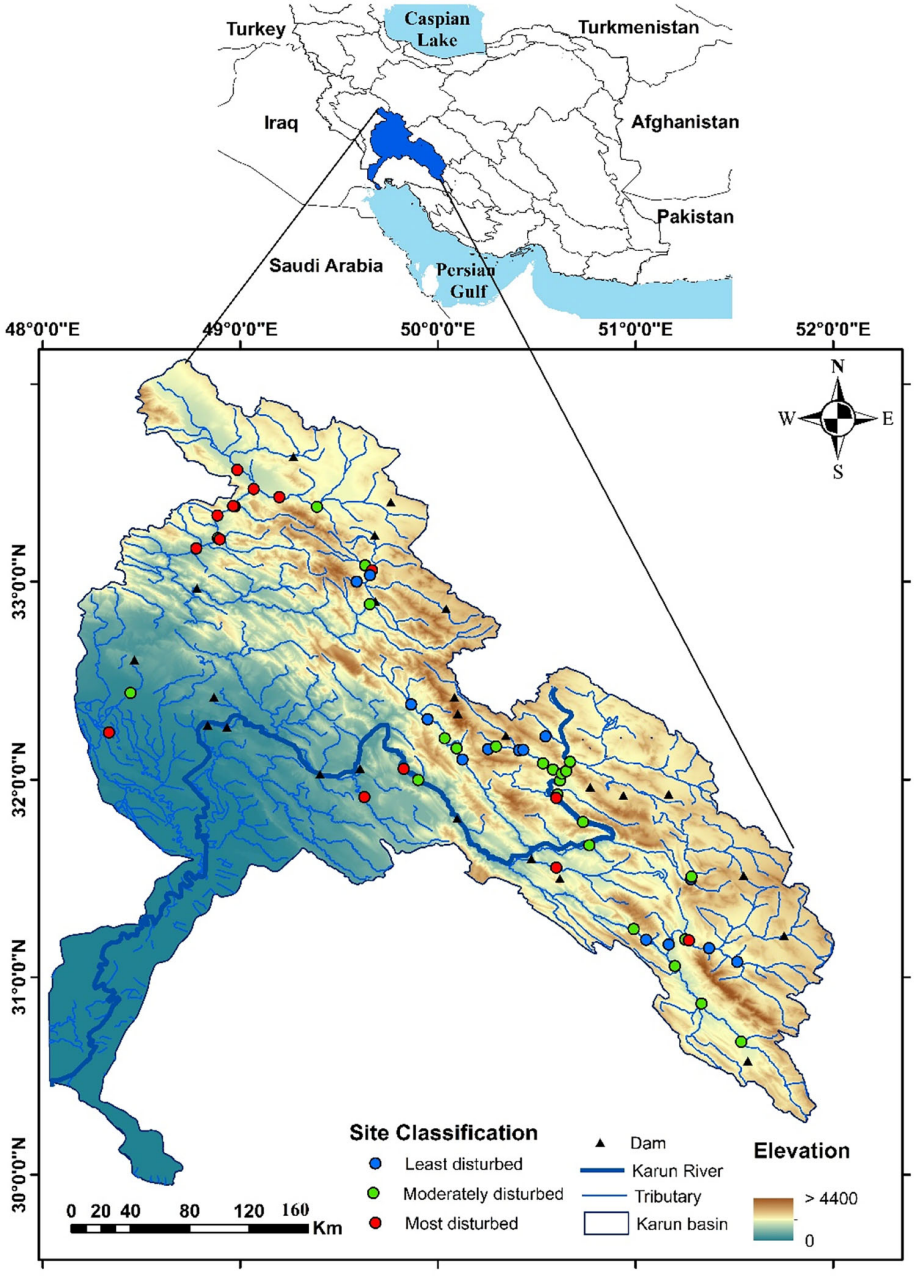
Map of watersheds in Iran (above), and distribution of sampling sites and dams in the Karun River Basin, classified by disturbance level (least, moderately, and most disturbed) based on PCA results. Color shading represents the elevation gradient, and triangles represent dams

Sampling was carried out in the Karun River basin, Iran, from July to August 2019 (Fig. 1). This period was selected based on practical and biological considerations: it coincides with the presence of different size classes of fish, is considered less stressful for fish, provides the longest window during which most sites are safely accessible, and minimizes safety risks for the field teams (Barbour et al. 1999). The initial site selection was refined based on site accessibility, sampling team security, the distance between sampling sites, changes in the type of land use among sites, and the locations of possible point-source pollutants entering the river upstream of candidate sites. Ultimately, we identified and sampled 53 sites within the basin that captured a wide range of environmental conditions (Table 1) (Zare Shahraki et al. 2021; Fathi et al. 2022a; Zare Shahraki et al. 2022b; Esmaeili Ofogh et al. 2024).

**Table 1:** Summary statistics (mean ± SD) of environmental variables measured across sampling sites

Variables	Unit	Value
Width	m	38.20 ± 37.53
Depth	cm	45.92 ± 20.81
Channel slope	%	1.72 ± 1.25
Substrate size	cm	10.17 ± 6.75
Flow velocity	(cm/s)	34.26 ± 13.44
Elevation	(MASL)	1331.27 ± 13.44
Nitrate (NO_3_)	(mg/L)	6.84 ± 5.15
Nitrite (NO_2_)	(mg/L)	0.09 ± 0.22
Biological oxygen demand (BOD)	(mg/L)	2.95 ± 1.06
Total phosphate (TP)	(mg/L)	1.31 ± 1.46
Total nitrogen (TN)	(mg/L)	8.35 ± 7.05
Dissolved oxygen (DO)	(mg/L)	8.4 ± 0.97
Chemical oxygen demand (COD)	(mg/L)	15.62 ± 11.17
Electrical conductivity (EC)	(μmho/cm)	841.96 ± 1446.54
Hardness	(mg/l caco3)	238.39 ± 172.51
Alkalinity	(mg/l caco3)	163.25 ± 50.100
Total coliforms	(n/100 ml)	2970.42 ± 4288.19
Total solids	(mg/L)	981.73 ± 1328.40
Turbidity	(mg/L)	38.66 ± 111.04
Total habitat score	-	128.34 ± 25.64
Instream score	-	42.15 ± 10.18
Morphological score	-	50.45 ± 13.27
Riparian score	-	33.34 ± 12.05

### Physicochemical and Physical Habitat Measurement

We collected water samples in triplicates using pre-washed plastic containers treated with 2% hydrochloric acid (HCl) (Fathi et al. 2022b, a). Before sampling, we rinsed the containers with river water to remove any contaminants. We transported the samples in cooling boxes to the laboratory. Water chemistry analyses were performed within 24 hours of collection following established protocols (Rice et al. 2017). At each sampling site, we determined physical characteristics, including river width, depth, channel slope, water velocity, high water mark, substrate size, vegetation protection, and the presence of dams (Barbour et al. 1999, Zare Shahraki et al. 2021, 2022a, 2022b). We evaluated physical habitat quality through visual assessment of ten parameters across three categories (instream features, channel morphology, and riparian/bank condition), with each parameter scored independently by five field experts on a 0–20 scale (Barbour et al. 1999). The instream score represented the summation of epifaunal substrate availability, embeddedness and velocity/depth combinations scores. Channel morphology scores were calculated by summing sediment deposition, channel flow status, channel alteration, and frequency of riffles (or bends), while riparian/bank condition scores combined bank stability, bank vegetative protection, and riparian vegetation zone width assessments. Total habitat quality was determined as the sum of these three component scores (Appendix A).

### Macroinvertebrate and Fish Sampling

At each site, fish and macroinvertebrate sampling were carried out simultaneously by respective teams. To ensure representative sampling across varying depths and habitat types, we sampled macroinvertebrates at ten transects distributed along a 200-meter river section using a Surber sampler in shallow habitats (<50-cm depth, 0.09 m^2^, 250 μm mesh), and a D-frame dip net in deeper habitats (>50-cm depth, 0.09 m^2^, 250 μm mesh) (Fathi et al. 2022a, b). After inspection and removal of large organic and inorganic debris, we combined all subsamples from a site into a single composite sample representative of the site. We preserved samples in 4% formalin for a maximum of 30 days before transferring to ethanol for laboratory analysis. We counted and identified macroinvertebrates to genus using taxonomic keys (Peckarsky et al. 1990; Tachet et al. 2010; Kriska, 2013; Bouchard 2021).

We sampled fish with a backpack electrofisher (Samus 1000) moving over a minimum distance of 200 meters (Zare Shahraki et al. 2022b, 2022a, 2023), covering both riverbanks and the full stream width along the reach. We collected fish samples from all available habitats (e.g., pools, riffles, and runs) and composited them into a single sample representative of the site (Zare Shahraki et al. 2022a, 2022b). To ensure sampling accuracy, we used a species detection curve and recorded the number of species caught during several fishing attempts (Fisher et al. 1943). We continued sampling until repeated efforts yielded no change in the species count (Zare Shahraki et al. 2024). We standardized catch per unit effort (CPUE) on distance rather than time because the time required to safely navigate the stream habitats varied greatly among sites (Flotemersch et al. 2011). We identified fish to species according to available keys and counted them in the field (Coad, 2020; Froese and Pauly, 2019; Jouladeh Roudbar et al. 2020; Jouladeh Roudbar et al. 2023a, 2023b). We returned 60% of the specimens to the river. To confirm field identification and retain voucher samples, we preserved the remaining specimens in 10% formalin.

### Establishing Reference Conditions

Formulating a multimetric index requires establishing the reference condition using a set of sites with least- or minimal anthropogenic impact (Schoolmaster et al. 2012). We used the Least Disturbed Condition (LDC) approach using Principal Component Analysis (PCA), which identifies reference locations based on the highest-quality conditions in the basin (Stoddard et al. 2006). We applied transformations to the physicochemical and habitat variables to approximate normal distributions (Zare Shahraki et al. 2021). To minimize redundancy and improve efficiency, we excluded one variable when strong correlations (∣r∣ ≥ 0.7) were detected (Dormann et al. 2013). In each correlated pair, we retained the variable that provided a more integrative or representative measure of environmental conditions. We used the first principal component (PC1) from a companion study (Zare Shahraki et al. 2021) composed of 17 standardized and centered variables as the primary stressor gradient (Appendix A). Depending on PC1 stressor direction, we classified sites in the less-disturbed, inner quartiles, and most-disturbed quartiles as least-disturbed, moderately disturbed, and most-disturbed sites, respectively (Blocksom and Johnson 2009). For additional details on the definition and identification of the least disturbed sites within the Karun River basin, refer to Zare Shahraki et al. (2021, 2022b).

### Metric Calculation

We calculated 54 candidate fish metrics describing species richness and composition, migratory behavior, feeding, habitat, and reproductive strategies (Appendix B) (Zare Shahraki et al. 2022b). Additionally, we computed 363 candidate macroinvertebrate metrics, categorized into taxa richness, assemblage composition, tolerance/intolerance indices, functional assemblage composition, and functional diversity indices (Esmaeili Ofogh et al. 2023, 2024) (Appendix C). To construct trait-based metrics, we identified 15 functional traits linked to disturbance gradient responses and grouped them into life history, ecological preferences, morphological, and dispersal traits (Larson et al. 2021; Esmaeili Ofogh et al. 2024) (Appendix D). We sourced trait information from peer-reviewed studies (Usseglio-Polatera et al. 2000; Tomanova and Usseglio-Polatera 2007) and online sources (Schmidt-Kloiber and Hering 2015). Metrics based on species composition and richness are widely used in MMIs due to their sensitivity to environmental changes (Plafkin et al. 1989; Helson and Williams 2013; Akamagwuna et al. 2022). In this study, we also included trait-based metrics reflecting life history, ecological preferences, morphology, and dispersal, as they can offer more ecologically meaningful responses to disturbance gradients (Esmaeili Ofogh et al. 2024). The metrics used in this research, covering both fish and macroinvertebrates, were computed based on the calculations described in companion studies (Esmaeili Ofogh et al. 2024; Zare Shahraki et al. 2022b).

### Index Development

We followed a systematic approach to identify metrics sensitive to anthropogenic disturbances. We excluded metrics with a median value of zero. We then applied Spearman correlation analysis between candidate metrics and natural background variables using only reference sites and removed metrics that showed strong correlations ( | *p* | > 0.7) with natural gradients (including altitude, river channel slope, Strahler stream order, river wetted width). In a second step, we assessed correlations with anthropogenic stressors across all sites, retaining only those metrics that were significantly associated with anthropogenic induced stress (*p* ≤ 0.01). This approach allowed us to distinguish metrics that primarily responded to anthropogenic disturbance. Next, we evaluated metric discriminatory power using boxplots (Blocksom and Johnson 2009) and Kruskal-Wallis tests across least-, moderately, and most-disturbed site categories. Metrics with significant differences (*p* ≤ 0.01) and box-and-whisker plot scores of 2 or 3 were retained (i.e., neither median overlapping with the interquartile range [IQR]; i.e., 25th to 75th percentile range, of the other group: box-plot score of 2; or no overlap of IQRs at all: score of 3; (Zare Shahraki et al. 2022b)). The final set of metrics meeting all these criteria was considered core metrics for the candidate Karun Fish Macroinvertebrate Indices (KFMIs).

We then developed the KFMI by combining the selected core metrics. Initially, we standardized all metrics to a 0–10 scale for consistency (Minns et al. 1994; Hughes and Oberdorff 1998; Blocksom and Johnson 2009; Zare Shahraki et al. 2022b). Following the “best subsets” approach, we combined subsets of 4, 5, 6, 7, 8, or 9 metrics to derive a series of multimetric indices (MMIs) (Magee et al. 2017; Zare Shahraki et al. 2022b). Averaging scores for each combination and multiplying by 10 rescaled the index to a 100-point scale, ensuring clarity and consistency. We randomly selected metrics from the screened pool to generate indices and calculate statistics. This process was repeated 10,000 times for each number of metrics, providing a broad evaluation of potential combinations across both small and large metric sets (Blocksom and Winters 2006; Zare Shahraki et al. 2022b).

We analysed the sensitivity of the MMIs by measuring the proportion of the most disturbed sites with index scores below the fifth percentile of the least disturbed sites (Van Sickle 2010). We also computed the mean and maximum correlations among metrics within each index and analysed the mean and standard deviation of index scores from the least disturbed sites. Starting with 60,000 MMIs, we filtered for sensitivity and applied additional criteria, requiring a maximum correlation among metrics of < 0.70 and a mean correlation of < 0.50 (Van Sickle 2010). From the top-ranked MMIs, we selected the final KFMIs based on tits performance, interpretability, reliability, and suitability across varying environmental conditions (Zare Shahraki et al. 2022b).

### Index Validation

We examined the discriminatory power of the final index, specifically its ability to separate least-, moderately, and most-disturbed sites, through box-and-whisker plots. We calculated the Spearman correlation analyses between the final index scores and abiotic disturbance variables (physicochemical and habitat variables) to assess the effectiveness of KFMI in the Karun River basin. Furthermore, we calculated Discrimination Efficiency (DE) to quantify the degree of separation between the index distributions for reference and disturbed sites (Zare Shahraki et al. 2022b) (Eq. 1).

1 $${\rm{DE}}=100\times \frac{a}{b}$$

**Fig. 2:**
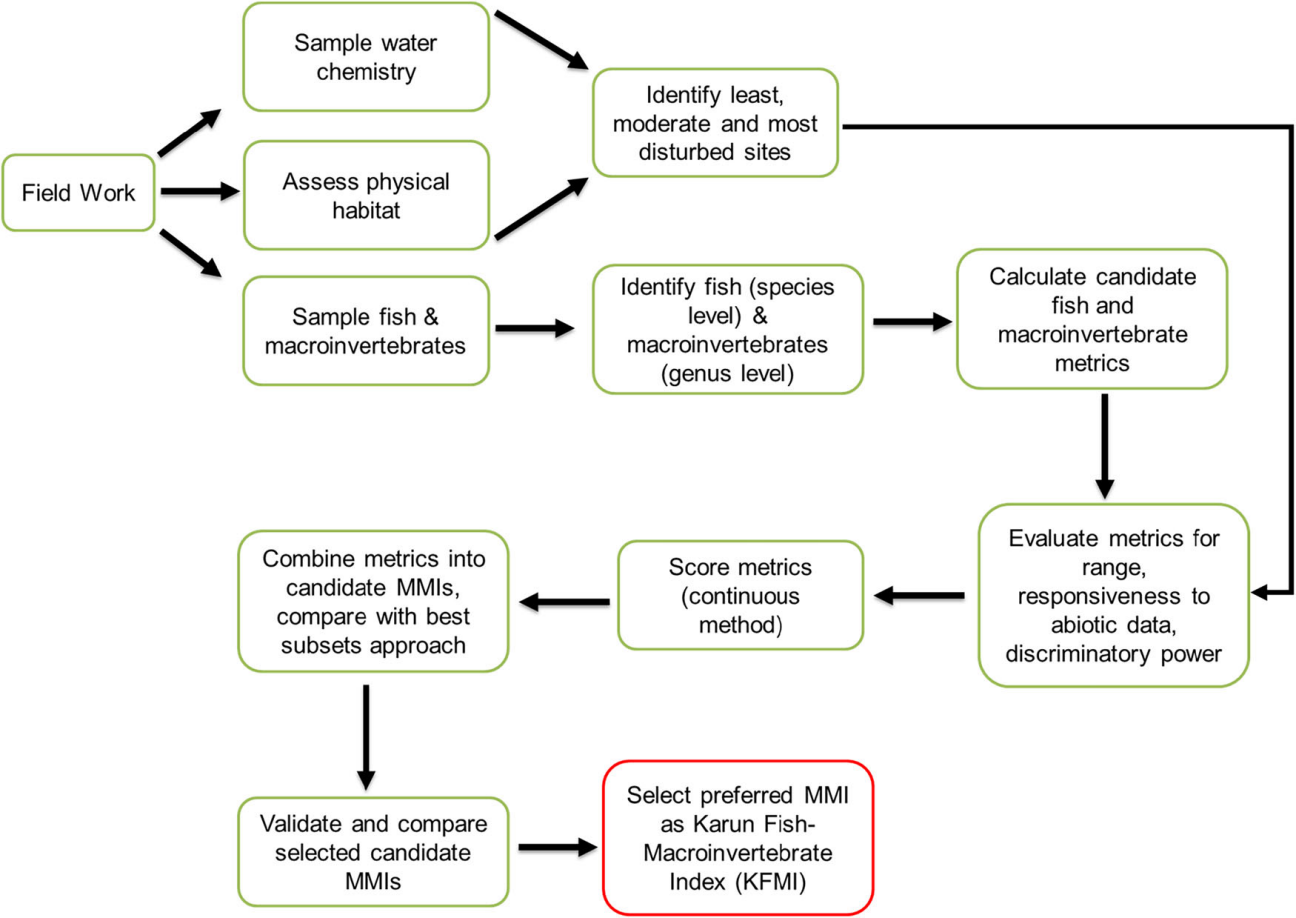
Stepwise development of the Karun Fish-Macroinvertebrate Index (KFMI), integrating fish and macroinvertebrate metrics

We calculated DE as the ratio of (a) the number of disturbed sites identified by both KFMI and PCA results to (b) the number of disturbed sites identified by PCA. A higher DE indicates that an index has stronger performance and a greater capacity to distinguish between disturbed and reference sites than PCA (Bressler et al. 2006). To assess the precision of the candidate KFMIs, we compared the coefficient of variation (CV) of KFMI values at reference sites. A lower CV indicates a more precise KFMI (Chen et al. 2014). We used linear regression to examine whether the variation in KFMI values at reference sites was systematically linked to background environmental conditions. Specifically, we analyzed the correlation between KFMI and three environmental descriptors: river wetted width, elevation, and river channel slope (Esmaeili Ofogh et al. 2023). After calculating the KFMI for all sites, we used the 5th and 25th percentiles of KFMI values from the least-disturbed sites as classification thresholds. The 5th percentile was used to distinguish the most-disturbed from moderately disturbed sites, and the 25th percentile to separate moderately disturbed from least-disturbed sites (Zare Shahraki et al. 2022b). We examined various statistics, including the range test, responsiveness analysis, and screening process of the best-performing KFMIs, and selected the most effective indices (Table 2). Last, we generated a scatter plot comparing KFMI site scores with the macroinvertebrate- and fish-based MMI scores. We conducted all statistical analyses using R software (v. 4.0.4, R Core Team incl. vegan (v. 2.6–4), and ggplot2 (v. 2.2.0) packages) and set at a level of significance of *p* = 0.01 unless stated differently. We followed a structured process to develop the KFMI (Fig. 2).

**Table 2:** Eigenvectors (loadings) of environmental variables for the first principal component of the PCA

Variables	PC1
Biological Oxygen Demand (BOD)	0.3991
**Total Phosphate (TP)**	**0.6555**
**Total Nitrogen (TN)**	**0.7196**
Dissolved Oxygen (DO)	−0.3119
Chemical Oxygen Demand (COD)	−0.0508
**Electrical Conductivity (EC)**	**0.8652**
**Hardness**	**0.6683**
**Alkalinity**	**0.7295**
**Total Coliforms**	**0.6312**
**Total Solids**	**0.9558**
**Turbidity**	**0.9369**
*Total Habitat Score	−**0.9036**
*Instream Score	−**0.4911**
*Morphological Score	−**0.9865**
*Riparian Score	0.4650
*% Clay	**0.6483**
*% Sand	**0.5800**

Parameters with an asterisk were calculated using the rapid bioassessment protocol (Barbour et al., 1999); bold rows indicate the influential variables in the study area

## Results

### Disturbance Classification

The principal component analysis (PCA) revealed that PC1 and PC2 explained 27 and 16% of the total variance, respectively. PC1 represented a gradient where sites with better total habitat and morphological scores appeared at the negative end, whereas sites with higher nutrients, clay, and sand occupied the positive end (Zare Shahraki et al. 2022b) (Table 2). From the total of 53 sites, 14 sites were classified as least-disturbed, 25 sites as moderately-disturbed, and 14 sites as most-disturbed (Zare Shahraki et al. 2021, 2022b).

### The Combined Multimetric Index Development and Validation

Sites 1, 16, and 30, where no fish were observed, were excluded from the analysis before metric calculation. Site 50 was removed from the dataset as it contained only a single fish species (*Hemiculter leucisculus*). Following screening, 15 fish metrics and 55 macroinvertebrate metrics were selected from the initial pools of metrics. The selection of final indices was based on the performance of retained metric subsets (Table 3). Two KFMIs with 7 metrics and one with 4 metrics indicated the best results. Among them, KFMI 7 d was selected as the best-performing index. Metrics and their respective scoring formulae in the KFMI 7 d are presented in Table 4. The core metrics in the final MMI exhibited a significant correlation with environmental variables (Appendix E). Our rationale for selecting KFMI 7 d over KFMI 4a, which had similar performance, is based on its inclusion of a greater number of metrics, which enhances its capacity to respond to a variety of disturbances. Furthermore, in comparison to KFMI 7b, KFMI 7 d exhibits a broader range of metrics derived from diverse categories, thereby amplifying its adaptability and comprehensive analytical potential because it accounts for different types of responses of the communities (e.g., taxonomic and functional responses; Esmaeili Ofogh et al. 2024).

**Table 3:** Performance evaluation of different multimetric indices (MMIs) using precision (CV), regression results (R²: proportion of least-disturbed site value variation linked to natural variables), and sensitivity (Discrimination Efficiency (DE))

MMIs	Metric	CV	R^2^			DE
			Slope	Altitude	Width	
KFMI 4a	▪ Relative abundance of edge inhabitant taxa ▪ Relative abundance (%) of Caenidae individuals ▪ Relative abundance (%) of Dugesiidae individuals ▪ Ephemeroptera + Plecoptera Taxa richness	0.07	0.030	0.010	0.00	94.7
KFMI 7 d	▪ Ephemeroptera + Plecoptera Taxa richness ▪ Relative richness of herbivorous taxa ▪ Total number of edge inhabitant taxa ▪ Relative abundance (%) of Caenidae individuals ▪ Relative abundance of lithophilic spawner taxa ▪ CWM of species with an aquatic adult life stage ▪ Relative abundance (%) of Dugesiidae individuals	0.07	0.002	0.00004	0.04	92.1
KFMI 7b	▪ Relative abundance of edge inhabitant taxa ▪ Sensitive Taxa richness (Taxa value 7-8-9-10) using BMWP TVs ▪ Relative richness of vegetative inhabitant taxa ▪ Relative abundance (%) of Caenidae individuals ▪ Relative abundance (%) of Dugesiidae individuals ▪ Relative abundance of Cyprinid taxa	0.06	0.002	0.290	0.05	92.1
MMI-macroinvertebrate	▪ Tolerant Taxa richness (Taxa value 1-2-3-4) using BMWP TVs ▪ Occurrential rarity ▪ Ephemeroptera + Plecoptera Taxa richness ▪ Relative abundance (%) of Ephemerellidae individuals ▪ CWM of species with rare/catastrophic propensity to drift	0.14	0.00	0.00	0.00	90.0
MMI-Fish	▪ Relative abundance of native and endemic species ▪ Relative richness of Herbivorous taxa ▪ Relative richness of Leuciscidae taxa ▪ Relative richness of migratory taxa ▪ Relative abundance of Cyprinid taxa ▪ Relative richness of vegetative inhabitant taxa ▪ Relative abundance of slow waterflow inhabitant taxa ▪ Total number of edge inhabitant taxa	0.04	0.234	0.048	0.011	81.6

**Table 4:** Metrics and formulae for scoring metrics included in the final Karun fish macroinvertebrate index (KFMI 7 d), where X represents the observed metric value

Metrics	Response to disturbances	Scoring formula
Relative richness of herbivorous fish taxa	Decrease	(X–9.22) / (38.87–9.22) × 100
Total number of edge inhabitant fish taxa	Increase	(4.1–X) / (4.1–0) × 100
Relative abundance of lithophilic spawner fish taxa	Decrease	(X–13.12) / (71.40–13.12) × 100
Ephemeroptera + Plecoptera taxa richness	Decrease	(X–2) / (10–2) × 100
Relative abundance (%) of Caenidae individuals	Increase	(11.55–X) / (11.55–0) × 100
Community Weighted Mean of species with an aquatic adult life stage	Increase	(0.15–X) / (0.15–0.002) × 100
Relative abundance (%) of Dugesiidae individuals	Increase	(4.87–X) / (4.87–0) × 100
**KFMI** = **Ʃ Metric scores × 1.42**		

**Fig. 3:**
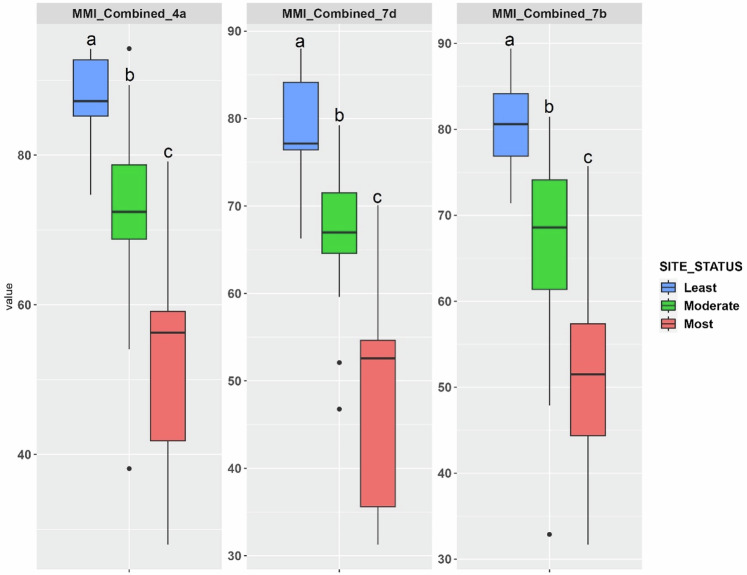
Best-performing KFMIs within various stress categories (least-, moderate, and most-disturbed sites). The letters above the boxes in each panel indicate statistically different groups (*p* < 0.01)

**Fig. 4:**
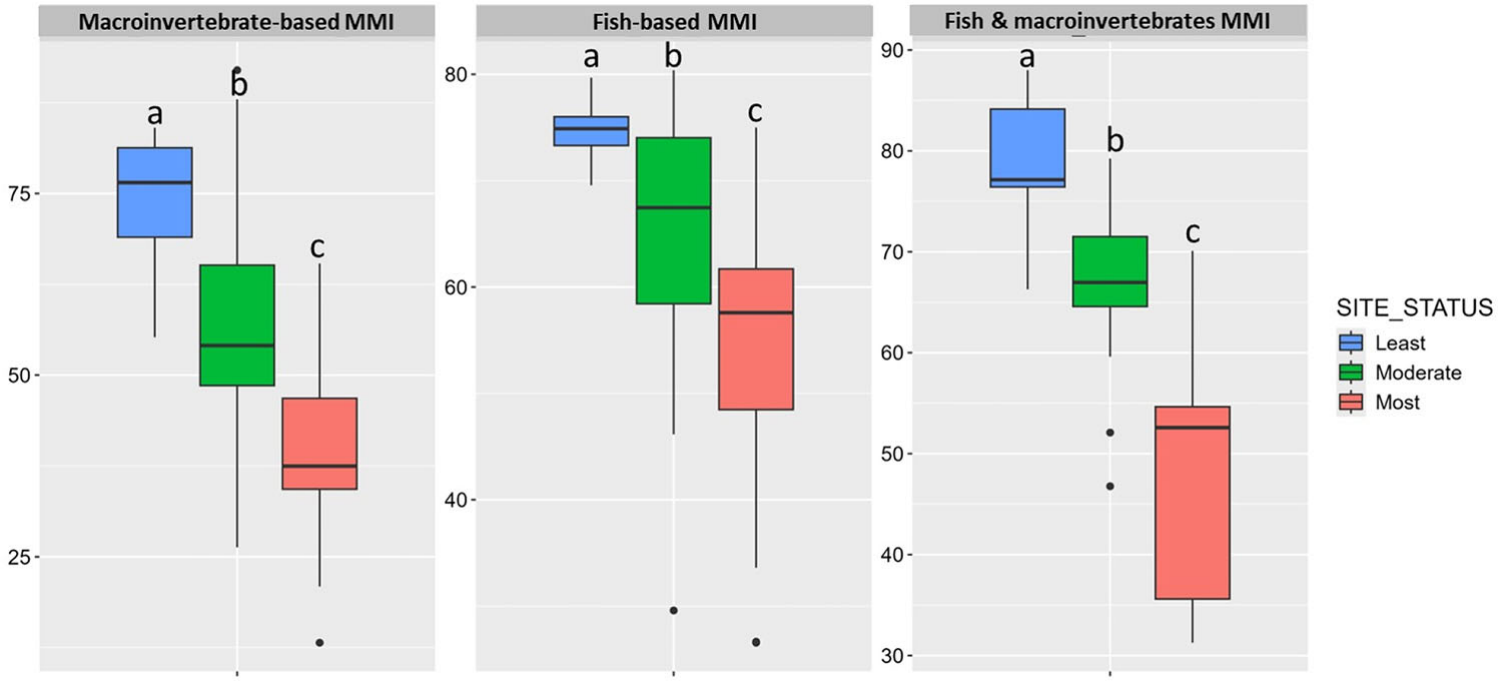
Comparing the best-performing KFMI (KFMI 7d) with macroinvertebrate-based (Esmaeili Ofogh et al. 2024) and fish-based (Zare Shahraki et al. 2022b) MMIs within various stress categories (least-, moderate, and most-disturbed sites). The letters above the boxes in each panel indicate statistically different groups (*p* < 0.01)

The effectiveness of the KFMI 7d was demonstrated by the significant correlations between the index scores and a range of environmental variables, including physicochemical parameters and habitat quality metrics (Table 5). All the remaining KFMIs were able to distinguish the least disturbed sites from moderate and most disturbed sites (Fig. 3). The application of the KFMI 7d identified 11, 10, and 28 sites as having good, moderate, and poor conditions, respectively. The CVs for the KFMIs in the reference sites ranged from 0.06 to 0.07 (Table 3). The regression test revealed that natural background variability did not significantly impact KFMI values (Table 3). The ability of the candidate index to distinguish reference from stressed sites based on DE ranged from 92.1% to 94.7%. To define ecosystem health using KFMI 7d, the index values were classified into three categories according to the 5th and 25th percentiles of KFMI values in the least disturbed sites: good (KFMI 76–100), moderate (KFMI 70–75), and poor condition (KFMI 0–69). Figure 4 compares the performance of the macroinvertebrate-based (Esmaeili Ofogh et al. 2024), fish-based (Zare Shahraki et al. 2022b), and combined multimetric indices (KFMI 7d) across site status categories. All three indices effectively distinguished between least, moderate, and most-disturbed sites, with the combined index exhibiting the greatest separation, suggesting enhanced sensitivity to environmental disturbance.

**Table 5:** Significant Spearman correlations between KFMI 7d and physicochemical and habitat-related environmental variables in the Karun River basin (*p* < 0.05)

Environmental variable	Correlation
EC	−0.48
Total phosphate	−0.29
Hardness	−0.49
Alkalinity	−0.50
Total Solid	−0.50
Turbidity	−0.42
Habitat Score	0.35
Morphological Score	0.52
Biological Oxygen Demand	−0.28
PC1	0.72

**Fig. 5:**
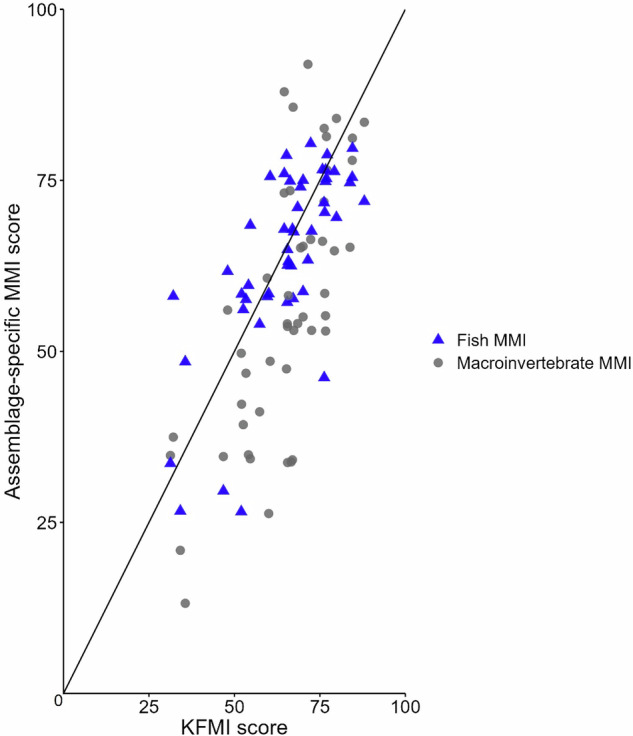
Scatter plot comparing site scores of the final combined multimetric index (KFMI 7d) with those of the fish-based MMI (blue triangles) and macroinvertebrate-based MMI (gray circles). The 1:1 line indicates agreement between KFMI and assemblage-specific MMIs. Points above the line indicate sites where the assemblage-specific MMI scored higher than the KFMI, while points below the line indicate the opposite

In Fig. 5, site scores (indicated by a triangle) below the 1:1 line had single assemblage MMI scores lower than the combined assemblage KFMI score, whereas site scores above the 1:1 line had single assemblage MMI scores higher than the combined assemblage KFMI score. Comparing KFMI site scores with the scores from the respective taxonomic groups (Fig. 5) indicated that for the fish MMI, there is a relatively even split above and below the 1:1 line along the gradient of conditions. However, for the macroinvertebrate MMI, more sites fall below the line indicating that the KFMI scored many sites higher than the macroinvertebrate MMI alone. Interestingly, this trend seems most evident at moderately scored sites. At the higher end of the scale, the macroinvertebrate MMI tends to score sites higher than the KFMI.

## Discussion

We developed and validated a new multimetric index (KFMI) that combines fish and macroinvertebrate metrics to assess the conditions of freshwater ecosystems in the Karun River basin. Our findings show that the combined index distinguishes more effectively between least, moderately, and most-disturbed sites than indices based on the respective taxonomic group used individualy. By incorporating biological responses from both fish and macroinvertebrates, we likely captured a broader range of ecological signals linked to anthropogenic disturbance. This improved sensitivity makes the KFMI a valuable tool for bioassessment in semi-arid river systems, where multiple stressors often interact and complicate ecological evaluations.

### The Core Metrics Composition of KFMI

The best-performing KFMI comprised seven metrics derived from fish and macroinvertebrate community composition, representing various metric categories such as taxa richness, community composition, functional diversity indices, functional feeding groups, reproduction status, and habitat preferences (Table 4). Like other MMIs combining these organism groups, the KFMI does not include equal numbers of metrics from fish and macroinvertebrate assemblages, (Pecl et al. 2017; Li et al. 2016; Ruaro et al. 2019). Whereas all included metrics were effective at detecting anthropogenic disturbance and distinguishing between reference and stressed sites, various studies have suggested that individual metrics are not always a good predictor of the overall performance of the combined index (Blocksom 2003; Van Sickle 2010). However, information provided by individual metrics can be of value by informing on specific disturbance types influencing overall index scores. Below, we discuss each metric of the final KFMI and specific attributes of the aquatic environment to which they are responsive.

In the final KFMI, two fish metrics were considered that were also included in the KFMMI, a fish-based MMI developed in a companion study (Zare Shahraki et al. 2022b). The first is the relative richness of herbivorous fish taxa which informs on overall ecosystem health. In disturbed conditions, the presence of fine sediment can lead to substrate instability preventing the accumulation and attachment of periphyton (Ruaro et al. 2019; Zare Shahraki et al. 2022b). This can lead to a decrease in food availability for herbivorous fish. In the current study, a significant negative correlation between the relative richness of herbivorous fish taxa is evident with turbidity, total suspended solids, and total solids (Appendix E). These conditions reduce the availability of resources for taxa that rely on periphyton as a food source (Cetra and Ferreira 2016; Ruaro 2019; Zare Shahraki et al. 2022b) and provide support for the importance of biotic interactions to understand and manage stressor effects on stream ecosystems (Bruder et al. 2017, 2019).

The second fish metric in the KFMI that was also in the KFMMI is the number of fish species with affinity to riparian habitats. In the current study, this metric demonstrated a significant negative correlation with the morphological score representing habitat conditions (Appendix E). This is in response to anthropogenic activities such as land use changes, urbanization, and habitat fragmentation that reduce the availability or quality of riparian habitat. As a result, this increased habitat heterogeneity provided by natural riparian habitats can offer opportunities for species to colonize or exploit new habitats (Allan 2004; Zare Shahraki et al. 2022a).

The third and final fish metric is lithophilic spawner fish taxa. These taxa depend on clean natural substrate for successful spawning. Anthropogenic disturbances such as land use changes, habitat fragmentation, and water pollution can cause alterations to the substrate of rivers and stream systems (Berkman and Rabeni 1987; Rabeni and Smale 1995), leading to a decline in lithophilic spawner abundance (Griffith et al. 2005; Vile and Henning 2018; Gatch 2019; Van Treeck et al. 2020). Consequently, lithophilic spawner taxa serve as excellent indicators of impairments caused for instance by siltation and channelization (Vile and Henning 2018; Wang et al. 2018; Gatch 2019; Van Treeck et al. 2020). Within the Karun River basin, a noteworthy positive correlation was also detected between the relative abundance of lithophilic spawner fish taxa and habitat scores (Appendix E).

Four macroinvertebrate metrics were included in the final KFMI. Ephemeroptera and Plecoptera taxa richness is sensitive to anthropogenic disturbances such as water pollution, habitat degradation, and hydromorphological alterations which can negatively affect the quality of their habitat and lead to declines in both the abundance and richness (Hershey and Lamberti 2001; Feld et al. 2015; Li et al. 2016; Suhaila and Che Salmah 2017). Hence Ephemeroptera and Plecoptera taxa richness is frequently used in multimetric indices (Paller and Specht 1997; Zhou et al. 2022). In the current study, this metric demonstrated a significant negative correlation to nutrient concentrations, substantiating the sensitivity of these taxa to nutrient enrichment. Additionally, a positive correlation was detected between Ephemeroptera and Plecoptera taxa richness and habitat scores, reflecting the positive influence of suitable habitat conditions on their occurrence and abundance (Appendix E).

The relative abundances of Caenidae and Dugesiidae families (Bressler et al., 2006; Jun et al., 2012), both included in the final KFMI, increased with intensifying anthropogenic disturbances. Caenidae, in particular, has operculate gills, which indicate an adaptation to silty substrates often found in disturbed environments (DeWalt et al. 2010 Juvigny-khenafou et al. 2021). Additionally, Caenidae are warm-water mayflies, and their abundance is often positively correlated with higher water temperatures (Kasangaki et al. 2008; Kibichii et al. 2015; Masese and Raburu 2017), suggesting that both anthropogenic disturbance and natural gradients such as temperature or elevation may shape their distribution. This dual response could potentially reduce their diagnostic power in a multimetric index, as increases in abundance may not exclusively indicate human pressure. Indeed, while Caenidae are often described as tolerant taxa (Dabessa et al. 2021; Tampo et al. 2021), no study has to our knowledge explicitly examined changes in their abundance along natural thermal or elevational gradients. Recognizing this knowledge gap, we evaluated candidate metrics against natural background variables using reference sites and excluded those strongly correlated with these gradients. Based on this screening, we found that Caenidae abundance responded consistently to disturbance and not to natural background variability, supporting its inclusion as a reliable metric in the final MMI.

Community-weighted mean (CWM) of macroinvertebrates with an aquatic adult life stage (e.g., Oligochaeta, Hirudinea, Prosobranchia, Tricladida, Heteroptera, and Coleoptera) was included in the KFMI because it increased with increasing anthropogenic disturbances. This result supports other studies that found high abundance of these families in polluted rivers and deteriorated habitats (Appendix E) (Hilsenhoff 1987; Tampo et al. 2020, 2021). The lifestyle of this group has been proposed as an explanation of their resistance to pollution (Kazancı et al. 2015) as opposed to members of the orders Ephemeroptera, Plecoptera, Trichoptera, and Odonata with their terrestrial adult life stages, which are often considered sensitive taxa (Kietzka et al. 2019; Tampo et al. 2021; Sripanya et al. 2022).

### Application of the KFMI for Stream Health Assessment

The final KFMI indicated good discrimination efficiency (92%) and precision in classifying sites into different ecological health categories and highlights the value of incorporating multiple biological assemblages in multimetric indices to support ecosystem assessment and management strategies. Moreover, only weak relationships were observed between KFMI scores and natural environmental variables (Table 3), indicating that these are not substantially influencing the selected metrics. Integrating both fish and macroinvertebrate metrics from different categories into a single index thus resulted in a comprehensive reflection of watershed conditions probably due to the specific sensitivities of each taxonomic group to diverse environmental gradients (Li et al. 2016; Ruaro et al. 2019). We observed the highest correlations between the KFMI and PC1 (including 17 physicochemical and habitat variables), suggesting that the KFMI provides a good measure of overall water and habitat quality (Table 5; Freund and Petty 2007; Li et al. 2016). This observation highlights the index’s capacity to detect and capture instream and near-stream anthropogenic disturbances.

When comparing KFMI site scores with those of the macroinvertebrate- and fish-based MMI scores (Fig. 5), it is important to remember that there does not exist an absolute benchmark score. The KFMI site scores in general moderate the scores derived from the independent MMIs and in doing so provide a more holistic assessment of biotic conditions. However, this could also be interpreted as the KFMI is masking information provided by the individual MMIs when viewed independently. Which approach is more useful for a specific application is therefore a management decision. If improved separation among site condition categories is desired, then there is a good argument for the use of the combined index score provided by the KFMI. If more specific stressor diagnosis is desired without the aid of additional analysis, then the preferred approach may be to consider the more specific information provided by the two taxon-specific MMIs separately. In both cases, identifying factors that are negatively affecting the condition can be enhanced by considering additional metrics that are diagnostic of specific stressors (Hering et al. 2006; Lemm et al. 2019; Jones et al. 2023; Rettig et al. 2023).

### Limitations and Repeatability

We conducted our sampling and calibration during July–August 2019 (dry season), so KFMI performance primarily reflects summer low-flow conditions rather than full seasonal dynamics. Because fish and macroinvertebrate assemblage structure and metric values vary across seasons, the class thresholds defined here may not hold outside the sampled period. We did not carry out replicate visits, which prevented us from quantifying site-level variance among seasons. The KFMI showed only weak associations with individual natural variables but correlated strongly with the composite disturbance axis (PC1; *r* = 0.72). Natural gradients may still interact with stressors and influence metric responses in ways that our study design could not fully disentangle. Therefore, we recommend that future applications repeat sampling across wet and dry seasons and across ecoregion and elevation bands to test temporal and spatial repeatability. We further suggest calibrating the index using band-specific thresholds (e.g., by elevation or ecoregion) or applying models that incorporate natural gradients when estimating metric weights and class boundaries.

## Conclusion

Freshwater ecosystems around the world and specifically in arid and semi-arid regions are increasingly threatened by land use change, hydrologic alteration, and declining water quality, which highlights the need for reliable tools to assess ecological conditions of freshwater ecosystems and guide restoration. To address this need, we developed the Karun Fish and Macroinvertebrate Index (KFMI), a multi-metric index that integrates complementary information from fish and macroinvertebrate assemblages. Our results show that KFMI reliably distinguished sites along a disturbance gradient and provides a robust basis for conservation planning and management decisions in the Karun River Basin.

As described above, our approach captures only a single season and does not account for variation across natural gradients. Future applications should extend the temporal and spatial coverage of the KFMI, evaluate its transferability to other basins, and where appropriate refine class thresholds using band-specific or model-based approaches. These efforts will support broader adoption in regional and national bioassessment programs while retaining and improving the KFMI’s practicality for data-limited, semi-arid river systems.

## Supplementary information

Appendices

## Data Availability

The data that support the findings of this study will be available after acceptance by the Journal in a publicly accessible repository based on the journal's suggestions.
