# Supplementary material for: Evaluating the Ecological Conditions of a Semi-Arid River Basin: A Multimetric Index Incorporating Fish and Macroinvertebrate assemblages
Source: Environ Manage. 2025 Nov 28;76(1):19. doi: 10.1007/s00267-025-02319-7 (PMC12662918; doi:10.1007/s00267-025-02319-7)
Supplement: Supplementary file 1 — Appendices [file 267_2025_2319_MOESM1_ESM.pdf]

Appendices

Appendix A. Measured physicochemical and physical habitat characteristics in the Karun River basin. Parameters marked with an asterisk were derived using the rapid bioassessment protocol (Barbour et al., 1999). Bold parameters were included in the PCA (This table is from our companion research (Zare Shahraki et al., 2021)).

Assessment category	Assessed features	Unit	Scale of measurement
**Physical habitat**	*River bank alteration	-	Reach
	*River channel alteration	-	
	*Vegetative protection	-	
	Riparian vegetative zone width	-	
	Channel Slope	Degree and percentage	
	Elevation	Meter above sea level	
	Width	cm	
	High water mark	m	
	Depth	cm	
	Reach length	m	
	Flow velocity	cm/s	
	Meso habitat types (% run, %riffle and %pool)	-<br>%	
	% Gravel	-	
	% Cobble		
	***% Clay & silt**		
	***% Sand**		
	Sediment deposition		
	***Total habitat score**		
	***Instream score**		
	***Morphological score**		
	***Riparian score**		
**Physico-chemical variables**	**Biological oxygen demand (BOD)**	(mg/L)	Site (Spot measurements)
	**Chemical oxygen demand (COD)**	(mg/L)	
	Escherichia coli (E. coli)	(n/100ml)	
	**Fecal coliform (FC)**	(n/100ml)	
	**Electrical conductivity (EC)**	(μmho/cm)	
	**Total hardness (TH)**	(mg/l caco3)	
	pH	-	
	**Total alkalinity (TA)**	(mg/l caco3)	
	Phosphate (PO_4)	(mg/L)	
	**Total phosphorus (TP)**	(mg/L)	
	Nitrate (NO_3)	(mg/L)	
	Nitrite (NO_2)	(mg/L)	
	Total ammonia nitrogen (TAN)	(mg/L)	
	Total kjeldahl nitrogen (TKN)	(mg/L)	
	**Total nitrogen (TN)**	(mg/L)	
	Total dissolved solids (TDS)	(mg/L)	
	Total suspended solid (TSS)	(mg/L)	
	**Total solid (TS)**	(mg/L)	
	**Turbidity (NTU)**	(mg/L)	
	Temperature (T)	°C	
	Oxygen saturation (DO %)	%	
	**Dissolved oxygen (DO)**	(mg/L)	

Appendix B. Candidate metrics for the development of KFMIs in the Karun River basin (This table is from our companion research (Zare Shahraki et al., 2022b)). *Bold indicates retained metrics after initial screening.

Metric	Metric Description
	**Richness and species composition**
1	Total number of individuals per site
2	Total number of taxa per site
3	Total number of families per site
4	Total number of Cyprinid taxa
5	**Relative abundance of Cyprinid taxa**
6	Relative richness of Cyprinid taxa
7	Total number of introduced taxa
8	Relative abundance of introduced taxa
9	Relative richness of introduced taxa
10	Total number of Leuciscidae taxa
11	Relative abundance of Leuciscidae taxa
12	**Relative richness of Leuciscidae taxa**
13	Total number of native and endemic taxa
14	**Relative abundance of native and endemic**
15	**Relative richness of native and endemic**
	**Functional feeding groups**
16	Total number of herbivorous taxa
17	Relative abundance of herbivorous taxa
18	**Relative richness of herbivorous taxa**
19	Total number of invertivorous taxa
20	Relative abundance of invertivorous taxa
21	Relative richness of invertivorous taxa
22	Total number of omnivorous taxa
23	Relative abundance of omnivorous taxa
24	Relative richness of omnivorous taxa
	**Reproduction status**
25	Total number of lithophilic spawner taxa
26	**Relative abundance of lithophilic spawner**
27	Relative richness of lithophilic spawner taxa
28	Total number of phytophilic spawner taxa
29	Relative abundance of phytophilic spawner
30	Relative richness of phytophilic spawner taxa
	**Migratory status**
31	Total number of migratory taxa
32	Relative abundance of migratory taxa
33	**Relative richness of migratory taxa**
	**Habitat preferences**
34	Total number of benthic taxa
35	Relative abundance of benthic taxa
36	Relative richness of benthic taxa

37	Total number of cold-water taxa
38	Relative abundance of cold-water taxa
39	Relative richness of cold-water taxa
40	**Total number of edge inhabitant taxa**
41	**Relative abundance of edge inhabitant**
42	**Relative richness of edge inhabitant taxa**
43	Total number of fast waterflow inhabitant taxa
44	Relative abundance of fast waterflow
45	Relative richness of fast waterflow inhabitant
46	Total number of rocky inhabitant taxa
47	Relative abundance of rocky inhabitant taxa
48	**Relative richness of rocky inhabitant taxa**
49	**Total number of slow waterflow inhabitant**
50	**Relative abundance of slow waterflow**
51	**Relative richness of Slow waterflow**
52	Total number of vegetative inhabitant taxa
53	Relative abundance of vegetative inhabitant
54	**Relative richness of vegetative inhabitant**

Appendix C. Candidate macroinvertebrate metrics used for the development of KFMIs in the Karun River basin. (This table is from our companion research (Esmaeili Ofogh et al., 2024, 2023)). *Bold indicates retained metrics after initial screening.

Metrics category	Metrics code	Description
Tolerance/ Intolerance	met1	Relative abundance (%) of Super-Tolerant individuals (Taxa values 9-10) using HFBI TVs
	met2	Super-Tolerant Taxa richness (Taxa value 9-10) using HFBI TVs
	met3	Relative percentage (%) of Super-Tolerant taxa (Taxa values 9-10) using HFBI TVs
	met4	Relative abundance (%) of Tolerant individuals (Taxa values 7-8-9-10) using HFBI TVs
	**met5**	**Tolerant Taxa richness (Taxa value 7-8-9-10) using HFBI TVs**
	**met6**	**Relative percentage (%) of Tolerant taxa (Taxa values 7-8-9-10) using HFBI TVs**
	met7	Relative abundance (%) of sensitive individuals (Taxa values 0-1-2-3) using HFBI TVs
	met8	Sensitive Taxa richness (Taxa value 0-1-2-3) using HFBI TVs
	met9	Relative percentage (%) of sensitive taxa (Taxa values 0-1-2-3) using HFBI TVs
	met10	Relative abundance (%) of super- sensitive individuals (Taxa values 0-1) using HFBI TVs
	met11	Super-sensitive Taxa richness (Taxa value 0-1) using HFBI TVs
	met12	Relative percentage (%) of super- sensitive taxa (Taxa values 0-1) using HFBI TVs
	met13	Relative abundance (%) of Super-Tolerant individuals (Taxa values 1-2) using BMWP TVs
	met14	Super-Tolerant Taxa richness (Taxa value 1-2) using BMWP TVs
	**met15**	**Relative percentage (%) of Super-Tolerant taxa (Taxa values 1-2) using BMWP TVs**
	met16	Relative abundance (%) of Tolerant individuals (Taxa values 1-2-3-4) using BMWP TVs
	**met17**	**Tolerant Taxa richness (Taxa value 1-2-3-4) using BMWP TVs**
	met18	Relative percentage (%) of Tolerant taxa (Taxa values 1-2-3-4) using BMWP TVs
	met19	Relative abundance (%) of sensitive individuals (Taxa values 7-8-9-10) using BMWP TVs
	**met20**	**sensitive Taxa richness (Taxa value 7-8-9-10) using BMWP TVs**
	**met21**	**Relative percentage (%) of sensitive taxa (Taxa values 7-8-9-10) using BMWP TVs**
	**met22**	**Relative abundance (%) of super- sensitive individuals (Taxa values 9-10) using BMWP TVs**
	**met23**	**Super-sensitive Taxa richness (Taxa value 9-10) using BMWP TVs**
	**met24**	**Relative percentage (%) of super- sensitive taxa (Taxa values 9-10) using BMWP TVs**
	met25	Hilsenhoff Family Biotic index
	met26	Biological Monitoring Working Party index
	**met27**	**Average Score Per Taxon index**
	**met28**	**Acid Water Indicator Community (AWIC) index**
	**met29**	**LIFE index**
	met30	PSI index
	met31	Whalley revised BMWP
	**met32**	**Whalley revised BMWP**
	**met33**	**WHPT presence-only ASPT**
	**met34**	**WHPT abundance weighted ASPT**
Taxonomic diversity indices	met35	Total abundance
	met36	Rarity Leory
	met37	Geographical rarity
	met38	Occurrential rarity
	met39	Total taxa richness
	met40	log Shannon-Wiener (S.W.LOG2)
	met41	ln Shannon-Wiener (S.W)
	met42	Simpson
	met43	Inverse Simpson (InvSimpson)
	met44	Brillouin
	met45	Margalef
	met46	Rényi entropy
	met47	Menhinick
	met48	McIntosh
	met49	Inverse Berger-Parker (InvB.P)
	met50	Hill numbers
	met51	Hill-Rényi
	met52	Hill-Tsallis
	met53	Simpson evenness (SimpsonE)

	met54	Pielou evenness (PielouE)
	met55	McIntosh evenness (McIntoshE)
	met56	Hill evenness (HillE)
	met57	Heip evenness (HeipE)
	met58	Camargo evenness (CamargoE)
	met59	Smith and Wilson’s Index (Evar)
	met60	Taxonomic diversity (D)
	met61	Taxonomic distinctness (Dstar)
	met62	Taxa richness of Archnidae
	**met63**	**Taxa richness of Insecta**
	met64	Taxa richness of Bivalvia
	**met65**	**Taxa richness of Gastropoda**
	met66	Taxa richness of Crustacea
	met67	Taxa richness of Hirudinae
	met68	Taxa richness of Oligochaeta
	met69	Taxa richness of Turbellaria
	met70	Taxa richness of Coloeptera
	met71	Taxa richness of Diptera
	**met72**	**Taxa richness of Ephemeroptera**
	met73	Taxa richness of Hemiptera
	met74	Taxa richness of Odonata
	**met75**	**Taxa richness of Plecoptera**
	met76	Taxa richness of Trichoptera
	met77	Taxa richness of Lamellibranchiata
	**met78**	**Taxa richness of Porosobranchiata**
	**met79**	**Taxa richness of Pulmonata**
	met80	Taxa richness of Tubificida
	met81	Taxa richness of Tricladida
	**met82**	**Ephemeroptera+Plecoptera+Trichoptera Taxa richness**
	**met83**	**Ephemeroptera+Plecoptera+Trichoptera+Odonata Taxa richness**
	**met84**	**Ephemeroptera+Plecoptera Taxa richness**
	met85	Hemiptera+Coloeptera+Diptera Taxa richness
	met86	Hemiptera+Coloeptera Taxa richness
Taxa richness	**met87**	**Ephemeroptera+Odonata+Trichoptera Taxa richness**
	met88	Oligocheata+Hirudinea Taxa richness
	met89	Odonata+Trichoptera Taxa richness
	met90	Oligocheata+Hirudinea+Turbellaria Taxa richness
	met91	Relative percentage of Taxa belonging to Archnidae
	**met92**	**Relative percentage of Taxa belonging to Insecta**
	met93	Relative percentage of Taxa belonging to Bivalvia
	**met94**	**Relative percentage of Taxa belonging to Gastropoda**
	met95	Relative percentage of Taxa belonging to Crustacea
	met96	Relative percentage of Taxa belonging to Hirudinea
	met97	Relative percentage of Taxa belonging to Oligochaeta
	met98	Relative percentage of Taxa belonging to Turbellaria
	met99	Relative percentage of Taxa belonging to Coleoptera
	met100	Relative percentage of Taxa belonging to Diptera
	met101	Relative percentage of Taxa belonging to Ephemeroptera
	met102	Relative percentage of Taxa belonging to Hemiptera
	met103	Relative percentage of Taxa belonging to Odonata
	**met104**	**Relative percentage of Taxa belonging to Plecoptera**
	met105	Relative percentage of Taxa belonging to Trichoptera
	met106	Relative percentage of Taxa belonging to Lamellibranchiata
	**met107**	**Relative percentage of Taxa belonging to Porosobranchiata**
	**met108**	**Relative percentage of Taxa belonging to Pulmonata**
	**met109**	**Relative percentage of Taxa belonging to Tubificida**
	met110	Relative percentage of Taxa belonging to Tricladida
	**met111**	**Relative percentage of Taxa belonging to Ephemeroptera+Plecoptera+Trichoptera**
	**met112**	**Relative percentage of Taxa belonging to Ephemeroptera+Plecoptera+Trichoptera+Odonata**

	**met113**	**Relative percentage of Taxa belonging to Ephemeroptera+Plecoptera**
	met114	Relative percentage of Taxa belonging to Hemiptera+Coloeptera+Diptera
	met115	Relative percentage of Taxa belonging to Hemiptera+Coloeptera
	met116	Relative percentage of Taxa belonging to Ephemeroptera+Odonata+Trichoptera
	**met117**	**Relative percentage of Taxa belonging to Oligocheata+Hirudinea**
	met118	Relative percentage of Taxa belonging to Oligocheata+Turbellaria
	met119	Relative percentage of Taxa belonging to Oligocheata+Hirudinea+Turbellaria
	met120	Total taxa richness of macroinvertebrates
Community composition	met121	Relative abundance (%) of Arachnidae individuals
	**met122**	**Relative abundance (%) of Insecta individuals**
	met123	Relative abundance (%) of Bivalvia individuals
	**met124**	**Relative abundance (%) of Gastropoda individuals**
	met125	Relative abundance (%) of Crustacea individuals
	met126	Relative abundance (%) of Hirudinae individuals
	**met127**	**Relative abundance (%) of Oligochaeta individuals**
	met128	Relative abundance (%) of Turbellaria individuals
	met129	Relative abundance (%) of Trombidiformes individuals
	met130	Relative abundance (%) of Coleoptera individuals
	met131	Relative abundance (%) of Colomba individuals
	met132	Relative abundance (%) of Diptera individuals
	met133	Relative abundance (%) of Ephemeroptera individuals
	met134	Relative abundance (%) of Hemiptera individuals
	met135	Relative abundance (%) of Hymenoptera individuals
	met136	Relative abundance (%) of Neuroptera individuals
	met137	Relative abundance (%) of Odonata individuals
	**met138**	**Relative abundance (%) of Plecoptera individuals**
	met139	Relative abundance (%) of Trichoptera individuals
	met140	Relative abundance (%) of Lamellibranchiata individuals
	met141	Relative abundance (%) of Porosobranchiata individuals
	**met142**	**Relative abundance (%) of Pulmonata individuals**
	met143	Relative abundance (%) of Amphipoda individuals
	met144	Relative abundance (%) of Isopoda individuals
	met145	Relative abundance (%) of Arhynchobdellida individuals
	met146	Relative abundance (%) of Rhynchobdellida individuals
	met147	Relative abundance (%) of Haplotaxida individuals
	met148	Relative abundance (%) of Lumbricida individuals
	met149	Relative abundance (%) of Lumbriculida individuals
	**met150**	**Relative abundance (%) of Tubificida individuals**
	met151	Relative abundance (%) of Tricladida individuals
	met152	Relative abundance (%) of Ephemeroptera+Plecoptera+Trchoptera individuals
	met153	Relative abundance (%) of Ephemeroptera+Plecoptera+Trchoptera+Odonata individuals
	met154	Relative abundance (%) of Ephemeroptera+Plecoptera individuals
	met155	Relative abundance (%) of Hemiptera+Coleoptera+Diptera individuals
	met156	Relative abundance (%) of Hemiptera+Coleoptera individuals
	met157	Relative abundance (%) of Ephemeroptera+Plecoptera+Trchoptera to Chironomidae individuals
	met158	Relative abundance (%) of Feltriidae individuals
	met159	Relative abundance (%) of Hygrobatidae individuals
	met160	Relative abundance (%) of Lebertiidae individuals
	met161	Relative abundance (%) of Limnocharidae individuals
	met162	Relative abundance (%) of Sperchontidae individuals
	met163	Relative abundance (%) of Torrenticolidae individuals
	met164	Relative abundance (%) of Unionicolidae individuals
	met165	Relative abundance (%) of Wettinidae individuals
	met166	Relative abundance (%) of Curculionidae individuals
	met167	Relative abundance (%) of Dryopidae individuals
	met168	Relative abundance (%) of Dytiscidae individuals
	met169	Relative abundance (%) of Elmidae individuals
	met170	Relative abundance (%) of Gyrinidae individuals
	met171	Relative abundance (%) of Haliplidae individuals

	met172	Relative abundance (%) of Helophoridae individuals
	met173	Relative abundance (%) of Hydraenidae individuals
	met174	Relative abundance (%) of Hydrophilidae individuals
	met175	Relative abundance (%) of Psephenidae individuals
	met176	Relative abundance (%) of Staphylinidae individuals
	met177	Relative abundance (%) of Isotomidae individuals
	met178	Relative abundance (%) of Anthomyiidae individuals
	met179	Relative abundance (%) of Athericidae individuals
	met180	Relative abundance (%) of Blephariceridae individuals
	met181	Relative abundance (%) of Ceratopogonidae individuals
	met182	Relative abundance (%) of Chironomidae individuals
	met183	Relative abundance (%) of Dixidae individuals
	met184	Relative abundance (%) of Empididae individuals
	met185	Relative abundance (%) of Ephydridae individuals
	met186	Relative abundance (%) of Limoniidae individuals
	met187	Relative abundance (%) of Psychodidae individuals
	met188	Relative abundance (%) of Rhagionidae individuals
	met189	Relative abundance (%) of Scathophagidae individuals
	met190	Relative abundance (%) of Simuliidae individuals
	met191	Relative abundance (%) of Stratiomyidae individuals
	met192	Relative abundance (%) of Tabanidae individuals
	met193	Relative abundance (%) of Tipulidae individuals
	met194	Relative abundance (%) of Thaumaleidae individuals
	met195	Relative abundance (%) of Baetidae individuals
	**met196**	**Relative abundance (%) of Caenidae individuals**
	met197	Relative abundance (%) of Ephemerellidae individuals
	**met198**	**Relative abundance (%) of Heptageniidae individuals**
	met199	Relative abundance (%) of Isonychiidae individuals
	met200	Relative abundance (%) of Leptophlebiidae individuals
	met201	Relative abundance (%) of Neoephemeridae individuals
	met202	Relative abundance (%) of Oligoneuriidae individuals
	met203	Relative abundance (%) of Potamanthidae individuals
	met204	Relative abundance (%) of Prosopistomatidae individuals
	met205	Relative abundance (%) of Corixidae individuals
	met206	Relative abundance (%) of Mesoveliidae individuals
	met207	Relative abundance (%) of Pleidae individuals
	met208	Relative abundance (%) of Heloridae individuals
	met209	Relative abundance (%) of Nemopteridae individuals
	met210	Relative abundance (%) of Aeshnidae individuals
	met211	Relative abundance (%) of Calopterygidae individuals
	met212	Relative abundance (%) of Coenagrionidae individuals
	met213	Relative abundance (%) of Cordulegasteridae individuals
	met214	Relative abundance (%) of Euphaeidae individuals
	met215	Relative abundance (%) of Gomphidae individuals
	met216	Relative abundance (%) of Lestidae individuals
	met217	Relative abundance (%) of Libellulidae individuals
	met218	Relative abundance (%) of Platycnemididae individuals
	met219	Relative abundance (%) of Capniidae individuals
	met220	Relative abundance (%) of Leuctridae individuals
	met221	Relative abundance (%) of Nemouridae individuals
	met222	Relative abundance (%) of Perlidae individuals
	met223	Relative abundance (%) of Perlodidae individuals
	met224	Relative abundance (%) of Taeniopterygidae individuals
	met225	Relative abundance (%) of Ecnomidae individuals
	met226	Relative abundance (%) of Hydropsychidae individuals
	met227	Relative abundance (%) of Hydroptilidae individuals
	met228	Relative abundance (%) of Leptoceridae individuals
	met229	Relative abundance (%) of Limnephilidae individuals
	met230	Relative abundance (%) of Philopotamidae individuals

	met231	Relative abundance (%) of Polycentropodidae individuals
	met232	Relative abundance (%) of Psychomyiidae individuals
	met233	Relative abundance (%) of Rhyacophilidae individuals
	met234	Relative abundance (%) of Margaritiferidae individuals
	met235	Relative abundance (%) of Sphaeriidae individuals
	met236	Relative abundance (%) of Unionidae individuals
	met237	Relative abundance (%) of Bithyniidae individuals
	met238	Relative abundance (%) of Hydrobiidae individuals
	met239	Relative abundance (%) of Melanopsidae individuals
	met240	Relative abundance (%) of Neritidae individuals
	met241	Relative abundance (%) of Potamididae individuals
	met242	Relative abundance (%) of Valvatidae individuals
	**met243**	**Relative abundance (%) of Viviparidae individuals**
	**met244**	**Relative abundance (%) of Lymnaeidae individuals**
	**met245**	**Relative abundance (%) of Planorbidae individuals**
	met246	Relative abundance (%) of Physidae individuals
	met247	Relative abundance (%) of Succineidae individuals
	met248	Relative abundance (%) of Gammaridae individuals
	met249	Relative abundance (%) of Asellidae individuals
	met250	Relative abundance (%) of Erpobdellidae individuals
	met251	Relative abundance (%) of Glossiphoniidae individuals
	met252	Relative abundance (%) of Piscicolidae individuals
	met253	Relative abundance (%) of Haplotaxidae individuals
	met254	Relative abundance (%) of Lumbricidae individuals
	met255	Relative abundance (%) of Lumbriculidae individuals
	met256	Relative abundance (%) of Naididae individuals
	met257	Relative abundance (%) of Tubificidae individuals
	**met258**	**Relative abundance (%) of Dugesiidae individuals**
	met259	Relative abundance (%) of Planariidae individuals
Functional diversity indices	met260	Community Weighted Mean (CWM) of species with Ovoviviparity reproduction strategy
	met261	CWM of species with Isolated eggs, free reproduction strategy
	met262	CWM of species with Isolated eggs, cemented reproduction strategy
	met263	CWM of species with Clutches, cemented or fixed reproduction strategy
	met264	CWM of species with Clutches, free reproduction strategy
	met265	CWM of species with Eggs or clutches, in vegetation (endophytic) reproduction strategy
	met266	CWM of species with Clutches, terrestrial reproduction strategy
	met267	CWM of species with Asexual reproduction strategy
	**met268**	**CWM of species with egg resistance form**
	met269	CWM of species with cocoon resistance form
	met270	CWM of species with cell resistance form
	met271	CWM of species with diapuse resistance form
	met272	CWM of species without resistance form
	met273	CWM of species with adult life span < 1 week
	met274	CWM of species with adult life span ≥ 1 week – 1 month
	met275	CWM of species with adult life span ≥ 1 month – 1 year
	met276	CWM of species with adult life span ≥ 1 year
	met277	CWM of species with No wings
	met278	CWM of species with 1 pair wing + halters
	met279	CWM of species with 1 pair wing + 1 pair of small hind wings
	met280	CWM of species with 1 pair wing+ 1 pair of elytra or hemielytra
	**met281**	**CWM of species with 2 similar-sized pairs**
	met282	CWM of species with streamlined/fusiform body shape
	met283	CWM of species with tubular body shape
	met284	CWM of species with dorsoventrally flattened body shape
	met286	CWM of species with round (humped) body shape
	met287	CWM of species with maximum body size ≤ 0.25 cm
	met288	CWM of species with maximum body size > 0.25- 0.5 cm
	met289	CWM of species with maximum body size > 0.5- 1 cm
	met290	CWM of species with maximum body size > 1- 2 cm

met291	CWM of species with maximum body size > 2- 4 cm
met292	CWM of species with maximum body size > 4- 8 cm
met293	CWM of species with maximum body size > 8 cm
met294	CWM of Flier species
met295	CWM of Surface swimmer species
met296	CWM of Swimmer species
met297	CWM of Crawler species
**met298**	**CWM of Interstitial (endobenthic) species**
met299	CWM of Interstitial (endobenthic) species
met300	CWM of Temporarily attached species
met301	CWM of Permanently attached species
met302	CWM of grazers/scrapers species
met303	CWM of miner’s species
met304	CWM of xylophagous taxa species
met305	CWM of shredders species
**met306**	**CWM of gatherers/collector’s species**
met307	CWM of active filter feeder’s species
met308	CWM of passive filter feeders species
met309	CWM of predator’s species
met310	CWM of parasites species
met311	CWM of other feeding types species
met312	CWM of species with life cycle duration ≤ 1
met313	CWM of species with life cycle duration >1
met314	CWM of species with < 1 potential life cycle per year
met315	CWM of species with 1 potential life cycle per year
met316	CWM of species with > 1 potential life cycle per year
met317	CWM of species with Egg aquatic life stage
met318	CWM of species with Larva aquatic life stage
met319	CWM of species with Pupa aquatic life stage
**met320**	**CWM of species with an aquatic adult life stage**
met321	CWM of species using Tegument for respiration
met322	CWM of species using Gill for respiration
met323	CWM of species using Plastron for respiration
met324	CWM of species using Spiracle (aerial) for respiration
**met325**	**CWM of species with Aquatic passive dispersal strategy**
met326	CWM of species with Aquatic active dispersal strategy
met327	CWM of species with Aerial passive dispersal strategy
met328	CWM of species with Aerial active dispersal strategy
met329	CWM of species with Rare/catastrophic propensity to drift
met330	CWM of species with Occasional propensity to drift
met331	CWM of species with Frequent propensity to drift
met332	CWM of species occurring only in standing waters
met333	CWM of species preferably occurring in standing waters; avoids current; rarely found in slowly flowing streams
**met334**	**CWM of species preferably occurring in standing waters but regularly occurring in slowly flowing streams**
met335	CWM of species usually found in streams; prefers slowly flowing streams and lentic zones; also found in standing waters
met336	CWM of species occurring in streams; prefers zones with moderate to high current
met337	CWM of species occurring in streams; bound to zones with high current
met338	CWM of species with no preference for a certain current velocity
met339	Functional richness calculated using all traits
met340	Functional dispersion calculated using all traits
met341	Functional evenness calculated using all traits
met342	Rao's quadratic entropy calculated using all traits
met343	Functional redundancy calculated using all traits
met344	Functional richness calculated using dispersal traits
met345	Functional dispersion calculated using dispersal traits
met346	Functional evenness calculated using dispersal traits

	met347	Rao's quadratic entropy calculated using dispersal traits
	met348	Functional redundancy calculated using dispersal traits
	met349	Functional richness calculated using life history traits
	met350	Functional dispersion calculated using life history traits
	**met351**	**Functional evenness calculated using life history traits**
	met352	Rao's quadratic entropy calculated using life history traits
	met353	Functional redundancy calculated using life history traits
	met354	Functional richness calculated using ecological traits
	met355	Functional dispersion calculated using ecological traits
	met356	Functional evenness calculated using ecological traits
	met357	Rao's quadratic entropy calculated using ecological traits
	met358	Functional redundancy calculated using ecological traits
	met359	Functional richness calculated using morphological traits
	met360	Functional dispersion calculated using morphological traits
	met361	Functional evenness calculated using morphological traits
	met362	Rao's quadratic entropy calculated using morphological traits
	met363	Functional redundancy calculated using morphological traits

Appendix D. Macroinvertebrate trait categories used in this study for calculation of functional information-based metrics. (This table is from our companion research (Esmaeili Ofogh et al., 2024, 2023)).

Trait category	Trait name	Trait modalities	Code
Life history traits	Aquatic life stage	Egg	EGG
		Larva	LARVA
		Pupa	NYMPH
		Adult	IMAGO
	Potential number of reproductive cycles per year (Voltinism)	< 1	SEMIVO
		1	UNIVO
		> 1	PLURIVO
	Life cycle duration	≤ 1	NCREQ1
		>1	NCRMO1
	Reproduction	Ovoviviparity	rep1
		Isolated eggs, free	rep2
		Isolated eggs, cemented	rep3
		Clutches, cemented or fixed	rep4
		Clutches, free	rep5
		Eggs or clutches, in vegetation (endophytic)	rep6
		Clutches, terrestrial	rep7
		Asexual reproduction	rep8
	Resistance forms	Egg	rf1
		Cocoon	rf2
		Cell	rf3
		Diapause	rf4
		None	rf5
Morphological traits	Maximum body size (cm)	< 0.25	SIZE1
		≥ 0.25–0.5	SIZE2
		≥ 0.5–1	SIZE3
		≥ 1–2	SIZE4
		≥ 2–4	SIZE5
		≥ 4–8	SIZE6
		≥ 8	SIZE7
	Body shape	Streamlined/fusiform	bs1
		Tubular	bs2
		Bluff(blocky)	bs3
		Dorsoventrally flattened	bs4
		Round (humped)	bs5
	Respiration	Tegument	Res1
		Gill	Res2
		Plastron	Res3
		Spiracle (aerial)	Res4
		Hydrostatic vesicle (aerial)	Res5
Ecological traits	Current preference	limnobiont	lib
		limnophil	lip
		limno- to rheophile	lrp
		rheo- to limnophil	rlp
		rheophile	rhp
		rheobiont	rhb
		indifferent	ind
	Feeding habits	grazers/scrapers	GRA
		miners	MIN
		xylophagous taxa	XYL
		shredders	SHR
		gatherers/collectors	GAT
		active filter feeders	AFF
		passive filter feeders	PFF
		predators	PRED

		parasites	PAR
		other feeding types	OTHER
	Locomotion and substrate relation	Flier	FLIER
		Surface swimmer	SURSWI
		Swimmer	SWIMM
		Crawler	CRAW
		Burrower (epibenthic)	BURR
		Interstitial (endobenthic)	INTST
		Temporarily attached	TEMATT
		Permanently attached	PERATT
**Mobility traits**	Dispersal strategy	Aquatic active	AQUACT
		Aquatic passive	AQUPAS
		Aerial active	AERACT
		Aerial passive	AERPAS
	Propensity to drift	Rare/catastrophic	drift1
		Occasional	drift2
		Frequent	drift3
	Adult life span	< 1 week	life1
		≥ 1 week – 1 month	life2
		≥ 1 month – 1 year	life3
		≥ 1 year	life4
	Wing pair type	No wings	wnb1
		1 pair + halters	wnb2
		1 pair + 1 pair of small hind wings	wnb3
		1 pair + 1 pair of elytra or hemelytra	wnb4
		2 similar-sized pairs	wnb5

Appendix E. Spearman correlation values between core metrics in the final MMI and environmental variables.

Core metrics in the final KFMI	Column	Correlation value
**Macroinvertebrate Metrics**		
Ephemeroptera + Plecoptera Taxa richness	NO_2	-0.334
	DO	0.344
	EC	-0.571
	Hardness	-0.352
	No_3	-0.409
	Alkalinity	-0.401
	TS	-0.388
	TN	-0.424
	TDS	-0.420
	PC1	0.686
	Habitat Score	0.469
	Water temperature	-0.474
	Morphological	0.541
Relative abundance (%) of Caenidae individuals	NO_2	0.365
	pH	-0.349
	Depth	0.388
	Water velocity	-0.356
	Water temperature	0.579
	Habitat score	-0.542
	Instream score	-0.386
	Morphological score	-0.525
	Clay	0.345
	Sand	0.373
	PC1	-0.469
	Width	0.320
Relative abundance (%) of Dugesiidae individuals	NO_2	0.532
	EC	0.409
	No_3	0.450
	Alkalinity	0.432
	Total Coliform	0.417
	TN	0.448
	Ecoli	0.410
	PC1	-0.365
Community-weighted mean (CWM) with an aquatic adult life stage	NO_2	0.379
	EC	0.476
	No_3	0.513
	Hardness	0.435
	Alkalinity	0.338
	TS	0.414
	Total nitrogen	0.518
	TDS	0.531
**Fish Metrics**		
Relative richness of herbivorous fish taxa	Clay	-0.315
	Ecoli	-0.429
	TSS	-0.384
	Total Coliform	-0.408
	TKN	-0.506

	TS	-0.325
	Turbidity	-0.384
Total number of edge inhabitant fish taxa	TDS	0.395
	TSS	0.470
	TP	0.296
	EC	0.325
	TKN	0.393
	TS	0.477
	Turbidity	0.470
	Morphological Score	-0.441
Relative abundance of lithophilic spawner fish taxa	TAN	-0.682
	TDS	-0.455
	TSS	-0.699
	NO_2	-0.347
	TP	-0.370
	DO	0.700
	Morphological Score	0.771
	Instream score	0.600
	Habitat Score	0.460
